# LY6E regulates Pseudorabies virus early infection via impairing viral internalization

**DOI:** 10.1186/s13567-026-01835-6

**Published:** 2026-09-12

**Authors:** Tanveer Ali Fazlani, Tianxing Cao, Yichao Wang, Yi Wang, Asad Jahangir, Xing Chen, Fayu Yang, Haixue Zheng, Shilei Zhang, Zixiang Zhu

**Affiliations:** 1 State Key Laboratory of Animal Disease Control and Prevention, College of Veterinary Medicine, Lanzhou University, Lanzhou Veterinary Research Institute, Chinese Academy of Agricultural Sciences, Lanzhou, 730046 China; 2 Gansu Province Research Center for Basic Disciplines of Pathogen Biology, Lanzhou, 730046 China

**Keywords:** LY6E, host factor, PRV, internalization

## Abstract

Pseudorabies virus (PRV) causes Aujeszky’s disease, posing an emerging threat to the livestock industry and raising concern for human health. Host cells employ a wide range of intrinsic factors to combat viral infections; however, the host factors involved in restricting PRV entry remain poorly understood. In this study, using both human and porcine cell models, we identified a potent antiviral role of LY6E (lymphocyte antigen 6, family member E) in suppressing PRV infection. Overexpression of LY6E resulted in a significant reduction in PRV infection, whereas depletion of LY6E increased infection, supporting a promising antiviral role against PRV. Structure–function analyses identified that the proper structure of LY6E is indispensable for its full antiviral activity. Transcriptomic analysis revealed that LY6E modulates specific host signaling pathways during PRV infection without triggering global transcriptional alterations. Furthermore, LY6E was found to interact with PRV envelope glycoproteins, implying its role in regulating viral entry. Subsequent investigations revealed that LY6E does not interfere with the initial attachment of PRV to host cells but specifically impairs viral internalization. Collectively, this study identifies LY6E as a previously unrecognized host restriction factor for PRV and highlights the role of surface proteins in controling viral entry. Additionally, it broadens the antiviral spectrum of LY6E to DNA viruses, beyond its previously reported host–viral interaction in RNA viruses.

**Supplementary Information:**

The online version contains supplementary material available at https://doi.org/10.1186/s13567-026-01835-6.

## Introduction

Host cells encode a wide range of intrinsic antiviral factors, which deploy defense mechanisms during viral infection at multiple stages of the replication cycle [1, 2]. Among these factors, proteins that control viral entry are important for the first line of defense [3]. Viral entry is a sophisticated, multi-step process that comprises attachment, internalization, membrane fusion, and uncoating [4]. These restriction factors play a vital role in the prevention of viral infection prior to the entry of the viral genome into the intracellular environment. Host factors that modulate these initial events can influence on viral tropism, replication efficiency, and ultimately the pathological outcome of infection [5]. Therefore, understanding how host proteins regulate viral entry is a central question in the field of antiviral biology.

Interferons (IFNs) are key cytokines of innate antiviral immunity that induce the expression of broad-spectrum antiviral genes and trigger the host's defensive response to infection [6]. However, the restriction of viral infection is not limited to IFN-mediated pathways; intrinsic factors can directly control viral replication without involving IFN signaling [7]. Consequently, there is growing interest in understanding the role of host restriction factors in controlling viral infection, while the cellular mechanisms that modulate viral entry and the initial events of the replication cycle remain incompletely characterized. Lymphocyte antigen 6, family member E (LY6E), a member of the lymphocyte antigen 6 family, has recently been identified as a regulator of host–virus interactions, with reported roles in viral infection, cellular signaling, and immune regulation. Notably, LY6E possesses a dual role as it restricts as well as enhances viral infections [8]. Based on these findings, we hypothesized that LY6E restricts PRV infection by interfering with the entry events of the viral replication cycle.

 PRV, also known as suid herpesvirus 1 (SuHV1), causes Aujeszky’s disease, a highly contagious and fatal swine disease [9]. PRV can infect different animal species, and it has become a global zoonotic threat, as PRV infection has been reported in humans [10]. The cross-species transmission of PRV to humans is supported by compelling evidence, including the isolation of PRV strains from patients and its detection through specific PRV antibodies by an enzyme-linked immunosorbent assay (ELISA) kit or metagenomic next-generation sequencing [11–16]. These results provide evidence that PRV can cross species barriers, likely facilitated by its genetic adaptability. In addition to posing a significant public health risk, PRV inflicts substantial economic losses on the global livestock industry due to high mortality and trade restrictions [17]. Therefore, the development of effective therapeutic strategies to control PRV infection in pigs and mitigate associated public health hazards are urgently needed.

## Materials and methods

### Cells and viruses

Human HEK293T cells, human A549 cells, mouse immortalized bone marrow-derived macrophages of mouse (iBMDM), PK-15 cells, porcine vascular epithelial cells (PVEC), and Vero cells were maintained under the standard culture conditions at 37 ℃ with 5% CO_2_. All cells were cultured in Dulbecco’s modified Eagle’s medium (DMEM) (VivaCell, Biosciences, Shanghai, China) supplemented with 10% fetal bovine serum (FBS) (ExCell Bio, Shanghai, China; Cat. No. FSP500) and 1% penicillin–streptomycin (Gibco Laboratories, Carlsbad, CA, USA). PRV-GFP, a thymidine kinase (TK)-negative recombinant pseudorabies virus in which green fluorescent protein (GFP), driven by the CMV promoter, was inserted into the TK gene locus and herpes simplex virus type 1 (HSV-1) were preserved in our laboratory and were propagated in Vero cells. Viral stocks were titrated on Vero cells using the 50% tissue culture infectious dose (TCID_50_) assay [18].

### Plasmid construction

The plasmids pHAGE−3*Myc-PRV-gB, -gC, -gD, -gE, -gG, -gH, -gI, -gK, -gL, and -gM and LY6E were constructed by cloning the synthesized genes (GENEWIZ, Suzhou, China) coding for PRV-*gB*, -*gC*, -*gD*, -*gE*, -*gG*, -*gH*, -*gI*, -*gK*, -*gL*, -*gM* and *LY6E* fragments into the vector pHAGE−3*Myc-puro, and the coding sequences were fused to the sequence coding for the epitope tag Myc at the C-terminus [19]. The coding region of the human *LY6E* gene was amplified using primers harboring NotI and NdeI restriction sites (forward: 5′−TTC AGG TGTC GTG AAG CCG CAT GAA GAT CTT CTT GCC AG TGC T–3′, reverse: 5′– ATG GTC TTT GTA GTC CAT ATG GGG GCC AAA CCG CAG CAG–3′), followed by digestion and ligation. A Flag-tagged LY6E construct was generated from the original Myc-LY6E plasmid, which was cloned into the pHAGE–Flag expression vector [19]. For selected constructs, the pHAGE–Flag–vc155 fragment was fused to LY6E to increase its apparent molecular weight and facilitate protein detection by immunoblotting. A site-specific mutant of LY6E (L36A/N99A) was generated by substitution of key residues using PCR-based site-directed mutagenesis. Additionally, truncated mutants (loss of extracellular domain/LU domain and C-terminal truncation) were constructed via PCR-based mutagenesis using mutation-specific primers (listed in Additional file 1). All constructs were confirmed by DNA sequencing prior to use.

### LY6E overexpression and knockdown

For LY6E overexpression, cells were transfected with the indicated expression plasmids using jetPrime® transfection reagent (Polyplus-transfection SA, IIIkrich, France) according to the manufacturer’s protocol. An empty vector was included as a negative control in all experiments. PK-15/PVEC cell lines were transfected with the sus-LY6E construct. After transfection, cells were incubated at 37 ℃ for 24 h.

For LY6E knockdown, small interfering RNAs (siRNAs) targeting LY6E or a non-targeting negative control siRNA (siNC) were synthesized by Sangon Biotech (Shanghai) Co., Ltd. (Shanghai, China). The following siRNA duplex sequences were used in A549 cells: two siLY6E duplexes (5′–GCU UGA ACC AGA AGA GCA AUC TT–3′ and 5′–GAU UGC UCU UCU GGU UCA AGC TT–3′; 5′–GAA UCU CGU GAC AUU UGG CCA TT–3′ and 5′–UGG CCA AAU GUC ACG AGA UUC TT–3′) and one siNC duplex (5′–UUC UCC GAA CGU GUC ACG UTT–3′ and 5′–ACG UGA CAC GUU CGG AGA ATT–3′). siRNA duplexes were transfected into cells using Lipofectamine™ 2000 reagent (Invitrogen, Thermo Fisher Scientific, Waltham, USA) according to the manufacturer’s instructions.

### Antibodies and reagents

Tagged proteins were detected using a mouse anti-MYC tag monoclonal antibody (Proteintech Group, Rosemont, IL, USA; Cat. no. 60003-2-Ig), a mouse anti-Flag (DYKDDDDK) tag monoclonal antibody (binds to the FLAG® tag epitope; Proteintech Group; Cat. no. 66008-4-Ig), a mouse anti-β-tubulin monoclonal antibody (Proteintech Group; Cat. no. 66240-1-Ig), a rabbit anti-ATP1A1 polyclonal antibody (Proteintech Group; Cat. no. 14418-1-AP), horseradish peroxidase (HRP)-conjugated anti-mouse IgG secondary antibody (BIODRAGON, Suzhou, China; Cat. no. BF03001) and HRP-conjugated anti-rabbit IgG secondary antibody (BIODRAGON; Cat. no. BF03008). Restriction enzymes NotI (Cat. no. R3189L) and NdeI (Cat. no. R0111S) were purchased from New England BioLabs (Ipswich, MA, USA).

### Co-immunoprecipitation

HEK293T cells were co-transfected with Flag-LY6E and Myc-tagged PRV glycoproteins. At 24 h post-transfection, cells were washed with phosphate-buffered saline (PBS), harvested into 1.5-mL tubes and centrifuged at 500 rpm for 3 min at 4 ℃, following which the cells were lysed with RIPA lysis buffer and vortexed at 4 ℃ for 20 min. The lysates were centrifuged at 1000 rpm for 5 min at 4 ℃. Following centrifugation, the lysates were incubated with anti-c-Myc magnetic beads (MedChemExpress, Cat. no. HY-K0206, USA) for 2 h at 4 ℃. Following incubation, the immunocomplexes were washed at least four times and analyzed by western blotting.

To further confirm the interaction between LY6E and PRV glycoproteins, HEK293T cells were transfected with Flag-vc-LY6E or the corresponding empty vector. For infection-based co-immunoprecipitation (co-IP) experiments, cells expressing Flag-vc-LY6E were infected with PRV at 0.1 multiplicity of infection (MOI). Appropriate uninfected empty-vector and Flag-vc-LY6E controls were included. At 24 h post-infection (hpi), cells were washed with PBS and harvested into 1.5-mL tubes. The cells were then centrifuged at 500 rpm for 3 min at 4 ℃, lysed with RIPA lysis buffer, and vortexed at 4 ℃ for 20 min. The lysates were centrifuged at 1000 rpm for 5 min at 4 ℃. Following centrifugation, the lysates were incubated with anti-Flag beads (MedChemExpress; Cat. no. HY-K0207, USA) for 45 min at 4 ℃ to capture Flag-tagged LY6E and associated protein complexes. Following incubation, the immunocomplexes were washed four times and analyzed by western blotting.

### Western blot analysis

Cells were lysed in RIPA buffer supplemented with PMSF (Beyotime, Shanghai, China; Cat. no. ST505) and protease inhibitor cocktail (TargetMol Chemicals, Boston, MA, USA; Cat. no. C0001). Equal amounts of total protein were separated by sodium dodecyl sulfate-polyacrylamide gel electrophoresis (SDS-PAGE) and transferred onto nitrocellulose membranes. Membranes were blocked in 6% skim milk for 1 h and incubated overnight with primary antibodies against PRV-gB, LY6E (Flag or MyC), and β-tubulin, which served as a loading control. Membranes were washed with TBST (Tris-buffered saline with Tween-20) 6 times and incubated with HRP-conjugated secondary antibodies for 60 min at room temperature, and then washed again 6 times with TBST. Signals were visualized using an enhanced chemiluminescence detection reagent (Advansta, San Jose, CA, USA; Cat. no. R-03031-D25).

### RNA extraction and quantitative reverse transcription PCR

Total RNA was extracted using RNAiso Easy (Takara Bio Inc., Shiga, Japan) according to the manufacturer’s instructions. Complementary DNA (cDNA) synthesis was performed using a PrimeScript™ Fast RT eagent Kit with gDNA Eraser (Takara Bio Inc.; Cat. no. RR092A). Quantitative reverse transcription PCR (RT-qPCR) was performed using gene-specific primers for human LY6E, PRV UL27(gB), glyceraldehyde 3-phosphate dehydrogenase (GAPDH), and HSV-1 gB (listed in Additional file 2). Relative messenger RNA (mRNA) expression levels were calculated using the comparative (Ct) cycle threshold (2^−ΔΔCt^) method with GAPDH as reference gene.

### Virus infection

Plasmid- or siRNA-transfected cells were infected with PRV–GFP at a MOI of 0.01. Following viral adsorption (1 h at 37 ℃), cells were washed and incubated in fresh medium containing 2% FBS for the specified time points prior to sample collection.

### Viral titration

Culture supernatants and cell lysates were harvested from cells overexpressing LY6E or carrying empty-vector control. To release intracellular virus, the cell samples were subjected to three freeze–thaw cycles between -80 ℃ and room temperature. All samples were clarified by centrifugation, and the supernatants were subjected to 10-fold serial dilutions before being added to 96-well plates seeded with Vero cells. The plates were incubated at 37 ℃ for 72 h. Viral titers were determined by calculating the TCID_50_ based on the observation of cytopathic effects (CPE).

### Confocal immunofluorescence microscopy

HeLa cells were cultured and transfected with Flag-tagged LY6E plasmid. At 24 h post-transfection, cells were washed with PBS and fixed with 4% paraformaldehyde (PFA). The cells were then washed 3 times with PBS and treated with 0.5% Triton X-100. After further washing, the cells were blocked with 5% bovine serum albumin (BSA) in PBS prior to being probed with primary antibody Anti-Flag (Sigma-Aldrich, St. Louis, MO, USA) 1:1000 and subsequent secondary Alexa Fluor 488-conjugated anti-mouse antibody 1:1000 (Invitrogen, Thermo Fisher Scientific). The nuclei were labeled by 4′,6-diamidino-2-phenylindole (DAPI) staining.

### Transcriptomic analysis

Three biological replicates of HEK293T cells were cultured in 6-well plates and transfected with 1.5 µg of empty vector (EV) or LY6E plasmid. At 24 h post-transfection, cells were either infected with PRV-GFP at an MOI of 0.1 or left uninfected. Samples were collected at 24 hpi for total RNA extraction and RNA sequencing (RNA-seq). RNA-seq datasets generated during this study are available in the NCBI Sequence Read Archive (SRA) under accession number PRJNA1461001.

### Immunofluorescence microscopy

For PRV-GFP infection experiments, infected cells were imaged directly in the absence of fixation or antibody staining. Fluorescence images were acquired at the indicated time points using a fluorescence microscope. Immediately after imaging, the same samples were processed for RNA extraction for RT-qPCR or protein lysis for western blot analysis.

### Attachment and internalization assay

Cells were seeded in 12-well culture plates and transfected with 1 µg of empty vector or LY6E expression plasmid. At the time of infection, culture plates were placed on ice and prechilled for 20 min. All subsequent steps for attachment assay were performed at 4 ℃.

For virus attachment assays, cells were washed once with prechilled PBS and incubated with PRV-GFP at an MOI of 1, diluted in prechilled serum-free DMEM. Virus adsorption was carried out for 1 h at 4 ℃ with gentle rocking every 15 min to ensure uniform virus-cell contact. Following incubation, cells were washed 3 times with prechilled PBS to remove unbound virus. Cells were lysed immediately for genomic DNA extraction.

For the internalization assay, after the initial attachment step at 4 ℃, cells were washed once with prechilled PBS and then incubated with DMEM supplemented with 2% FBS at 37 ℃ for 2 h to allow viral internalization. Cells were subsequently washed once with citrate buffer (40 mM sodium citrate, 10 mM KCl, 135 mM NaCl, pH 3.0) to remove surface-bound but non-internalized virions, followed by additional washes with PBS. Cells were then collected for genomic DNA extraction.

Genomic DNA was extracted using a commercial kit (TIANGEN Biotech, Beijing, China; Cat. no. GDP304) according to the manufacturer’s instructions. Viral genome copy numbers were quantified by qPCR targeting the PRV-*gB* gene, with *GAPDH* as an internal control. Relative viral DNA copy numbers were calculated to assess attachment and internalization efficiencies.

### Statistical analysis

Data are presented as the mean ± standard error of the mean (SEM) from at least three independent biological replicates. Statistical analysis was performed using Student’s *t*-test for comparisons between two groups or one-way analysis of variance (ANOVA) followed by Tukey’s post hoc test for multiple comparisons. l Statistical significance was indicated as *P < 0.01 and **P < 0.001; ***P < 0.0001; ns, not significant.

## Results

### Broad antiviral effect of LY6E against PRV in multiple cell types

**Figure 1:**
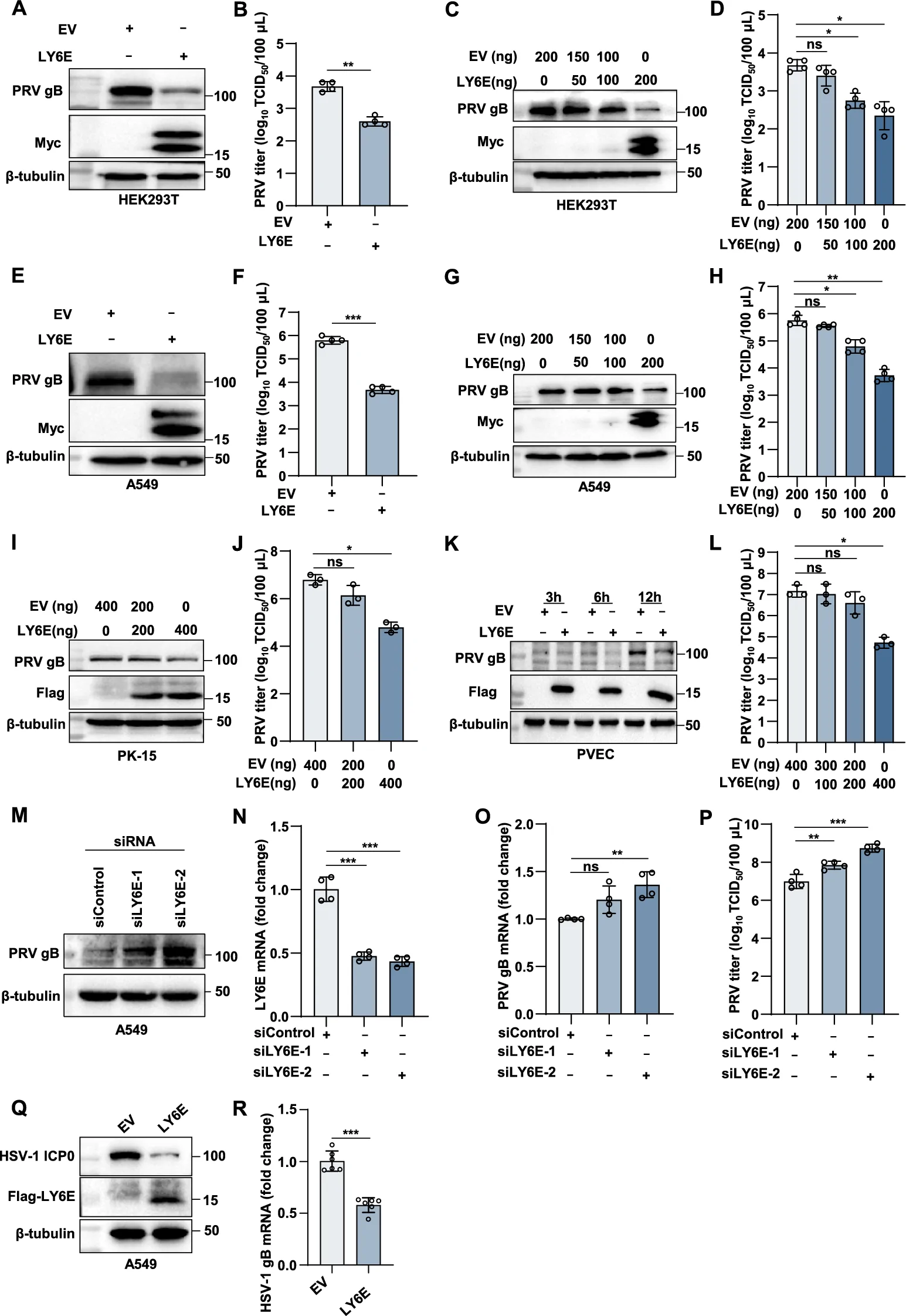
**Broad antiviral effect of lymphocyte antigen 6, family member E (LY6E) against Pseudorabies virus (PRV) in multiple cell types.** **A–D** HEK293T cells were transfected with empty vector (EV) or increasing doses of LY6E plasmid and infected with PRV. **A**, **C** Immunoblot analysis of LY6E and PRV-gB with β-tubulin as a loading control. **B**, **D** Viral titers in cell lysates and supernatants were quantified using the 50% tissue culture infectious dose (TCID_50_), showing dose-dependent reduction in PRV replication upon LY6E overexpression. **E–H** A549 cells were transfected with the EV or LY6E plasmid and infected with PRV. **E**, **G** immunoblot analysis of LY6E and PRV gB. **F**, **H** TCID_50_ quantification shows the reduced PRV titers in LY6E-overexpressing cells. **I, J** PK-15 cells were transfected with the LY6E plasmid and infected with PRV. **I** Immunoblot analysis of PRV-gB. **J** TCID_50_ shows reduced viral titers in LY6E-expressing cells compared with the EV control. **K, L** PVEC cells were transfected with the LY6E plasmid and infected with PRV. **K** Time-course immunoblot analysis of PRV-gB at the indicated time points. **L** Dose–response inhibition of PRV replication measured by TCID_50_ in PVEC cells transfected with increasing amounts of LY6E. **M–P** LY6E knockdown by small interfering RNA (siRNA) in A549 cells and infected with PRV. **M** Immunoblot analysis of PRV-gB with β-tubulin as a loading control. **N** Quantitative reverse transciption PCR (RT-qPCR) of LY6E mRNA relative to control siRNA. **O** RT-qPCR quantification of PRV transcripts following LY6E knockdown. **P** Viral titers in cell lysates and supernatants were quantified using the TCID_50_ assay following LY6E knockdown. **Q-R** A549 cells were transfected with EV or LY6E plasmid and infected with HSV-1 **Q** Immunoblot analysis of LY6E and herpes simplex virus type 1 (HSV-1) with β-tubulin as a loading control. **R** RT-qPCR quantification of HSV-1 gB messenger RNA (mRNA) expression. Cells were transfected with 200 ng plasmid EV or LY6E per wells for **A**, **B**, **E**, **F**, **Q**, **R** and 400 ng per well for **K**. For the siRNA experiments (**M**–**P**) cells were transfected in 12-well plates with 40 pmol of siRNA. Asterisks indicate a strong significant difference at *P < 0.01 and **P < 0.001 and a very strong statistical difference at ***P < 0.0001; ns, not significant.

The host immune response is primarily triggered by type I INF production during infection. As* LY6E* is an IFN-stimulated gene (ISG), we assessed the transcriptional response of LY6E to IFN-β stimulation in mouse iBMDMs. LY6E mRNA expression was significantly upregulated in a time-dependent manner following IFN-β treatment (Additional file 3). To determine its antiviral activity, we investigated the impact of LY6E overexpression on PRV replication in multiple cell types. HEK293T cells were transfected with LY6E or an empty vector and subsequently infected with PRV. Immunoblot analysis revealed that LY6E overexpression significantly decreased PRV-gB protein levels compared with vector-transfected control cells (Figure 1A). Similarly, PRV titers were markedly decreased in LY6E-overexpressing cells as quantified by TCID_50_ assay (Figure 1B). To evaluate the dose-dependent antiviral effect of LY6E, HEK293T cells were transfected with increasing doses of LY6E plasmid prior to PRV infection. Increasing the dose of LY6E led to a progressive reduction in PRV gB protein levels (Figure 1C), accompanied by a corresponding decrease in viral titers (Figure 1D), obviously demonstrating dose-dependent inhibition of PRV replication by LY6E. We further assessed the antiviral activity of LY6E in other cell lines. LY6E overexpression in A549 cells similarly inhibited PRV replication, as evidenced by a marked decrease in PRV-gB protein expression (Figure 1E, G) and a significant decrease in viral titers measured by TCID_50_ assay (Figure 1F, H). These findings suggest that the restrictions of PRV replication imposed by LY6E are not limited to HEK293T cells.

The inhibitory effect of LY6E on PRV was further confirmed in porcine PK-15 cells, a biologically relevant host cell type for PRV infection. Overexpression of LY6E in PK-15 cells resulted in decreased PRV-gB protein levels (Figure 1I) and diminished viral replication (Figure 1J), indicating that LY6E suppresses PRV infection across species. Moreover, PVECs, a cell type derived from the natural host, were transfected with LY6E and infected with PRV to examine the temporal dynamics of LY6E-mediated antiviral activity. Immunoblot analysis showed that LY6E-expressing cells exhibited a progressive, time-dependent decrease in PRV-gB protein levels (Figure 1K). Simultaneously, a dose-dependent decrease in PRV titers was observed with increasing doses of LY6E plasmid in PVECs, as measured with the TCID_50_ assay (Figure 1L), supporting the role of LY6E as a robust antiviral gene. Notably, the differential antiviral effects observed in human and porcine cell lines are likely to be influenced by differences in transfection efficiency as human cell lines show higher transfection efficiency as compared to porcine cell lines, which require a higher plasmid dose to achieve a comparable expression level (as shown in Additional file 4).

We used siRNA to knockdown LY6E in A549 cells, followed by PRV infection, to confirm our overexpression findings. Knockdown of LY6E by siRNA led to a considerable decline in LY6E mRNA levels (Figure 1N), concomitant with increased PRV-gB protein expression, mRNA levels, and PRV titers (Figure 1M, O, P). Collectively, these gain- and loss-of-function experiments validate that LY6E exerts a potent and conserved antiviral effect against PRV across multiple cell types and species. This antiviral activity functions in both dose- and time-dependent manners to suppress viral protein expression and the production of infectious virions. Furthermore, we determined that LY6E-mediated restriction extends to HSV-1; we examined HSV-1 infection in LY6E-expressing cells. LY6E overexpression significantly reduced HSV-1 infection, as indicated by reduced HSV-1 ICP0 protein in immunoblot and reduced HSV-1 gB mRNA levels (Figure 1Q, R). These findings suggest that LY6E exerts a broader antiviral effect against alphaherpesviruses.

### Site-specific mutation of critical residues L36 and N99 does not significantly impair LY6E-mediated antiviral restriction

**Figure 2:**
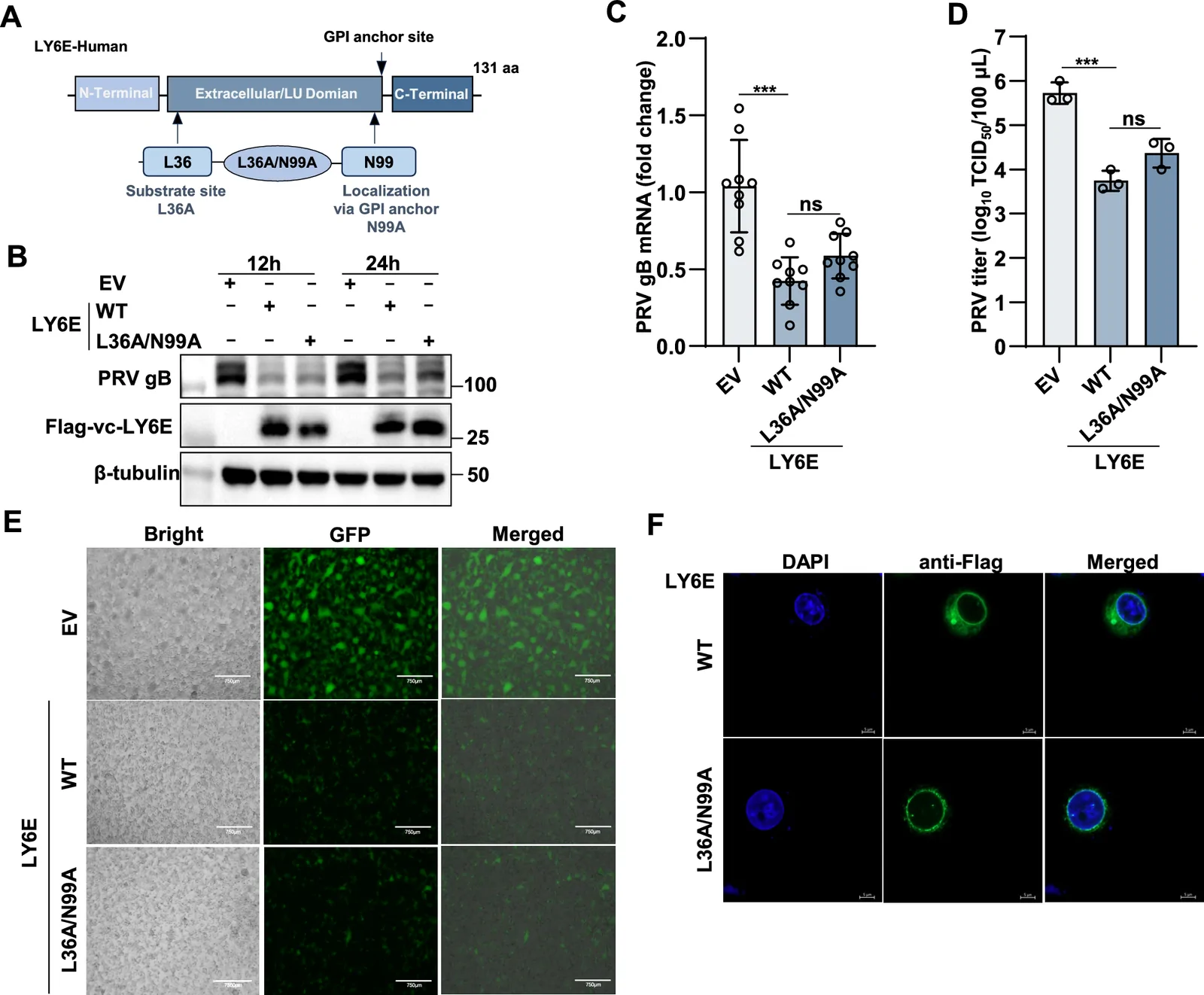
**Antiviral activity of site-specific lymphocyte antigen 6, family member E (LY6E) mutants against Pseudorabies virus (PRV).** **A** Schematic representation of LY6E protein, showing the extracellular/LU domain, C-terminal glycosylphosphatidylinositol (GPI) anchor site, and key residues. The L36A/N99A mutant construct is shown relative to wild-type (WT) LY6E. LY6E was fused to a truncated green fluorescent protein (GFP) fragment to increase molecular weight and facilitate detection. β-tubulin serves as a loading control. **B** Immunoblot analysis of PRV-gB expression in HEK293T cells transfected with empty vector (EV), WT LY6E, or L36A/N99A mutant and infected with PRV-GFP for 12 and 24 h. **C** Quantitative reverse transciption PCR (RT-qPCR) analysis of PRV-gB mRNA levels in cells expressing EV, WT LY6E, or L36A/N99A mutant. **D** Viral titers in culture supernatants measured by the 50% tissue culture infectious dose (TCID_50_) assay. **E** Immunofluorescence analysis of PRV infection. Presence of GFP indicates viral infection, and bright images show cell morphology and merged images show overlay of GFP and bright images. **F** Confocal immunofluorescence analysis of Flag-tagged WT-LY6E and L36A/N99A. LY6E was detected using anti-Flag antibody (green), and nuclei were counterstained with DAPI (blue). Asterisks indicates a very strong statistical difference at ***P < 0.0001; ns, not significant.

Members of the LY6/uPAR superfamily, including LY6E, typically contain ten cysteines that form a highly conserved, three-finger-fold motif through disulfide bonds and localize on the cell membrane via a glycosylphosphatidylinositol (GPI) anchor [20, 21]. Recent studies have revealed that LY6E promotes viral entry and restricts viral infection through conserved residues L36 and N99, which are required for proper membrane localization via GPI anchoring [22, 23]. To identify the structural factors underlying LY6E-mediated antiviral activity against PRV, we produced site-specific mutations at these two functionally important residues: L36 and N99. We constructed a site-specific double mutant (L36A/N99A) targeting these two residues and compared its antiviral activity with that of wild-type (WT) LY6E (Figure 2A).

To assess the functional role of the L36A/N99A mutation, HEK293T cells were transfected with WT LY6E, mutant LY6E, or EV, followed by PRV infection. Immunoblot analysis revealed that both WT LY6E and the L36A/N99A mutant significantly suppressed PRV-gB protein levels at 12 and 24 hpi compared with the EV control (Figure 2B). Consistent with this result, RT-qPCR confirmed significantly lower PRV-gB transcript abundance in cells expressing either WT LY6E or the L36A/N99A mutant relative to EV controls (Figure 2C). Viral titer measurements via TCID_50_ assays further validated these observations: both WT LY6E and the L36A/N99A mutant significantly suppressed PRV production (Figure 2D). To visualize the antiviral effect at the cellular level, we utilized PRV-GFP for fluorescence microscopy. GFP-positive signals (indicating PRV-infected cells) were significantly decreased in cells expressing either WT LY6E or the L36A/N99A mutant compared to vector-transfected cells, suggesting effective suppression of viral infection and demonstrating that mutation of L36/N99 residues does not impair LY6E-mediated antiviral activity against PRV (Figure 2E). Since the mutation of L36 and N99 residues did not significantly impair the inhibitory effect of LY6E against PRV, we further examined the subcellular localization of WT LY6E and L36A/N99A mutant (Figure 2F). By confocal microscopy, WT LY6E exhibited membrane-associated as well as intracellular patterns. The L36A/N99A mutant appeared to be more concentrated in the perinuclear region. Taken together, no statistically significant differences were observed between WT LY6E and the L36A/N99A mutant in antiviral assays performed in this study. These findings demonstrate that L36 and N99 residues are not essential for LY6E-mediated restriction of PRV. The confocal microscopy results suggested subtle differences in fluorescence distribution between WT LY6E and the L36A/N99A mutant; however, these observations are not sufficiently pronounced to support a conclusion regarding subcellular localization. Additional biochemical fractionation demonstrated that WT LY6E was predominantly associated with the membrane fraction, supporting its membrane-associated localization whereas a lower level of protein was detected in the cytosolic fraction (Additional file 5). Further studies will be required to determine whether L36 and N99 residues contribute to LY6E trafficking, intracellular distribution, or other aspects of its antiviral mechanism.

### Domain-specific contribution of LY6E to antiviral restriction of PRV

**Figure 3:**
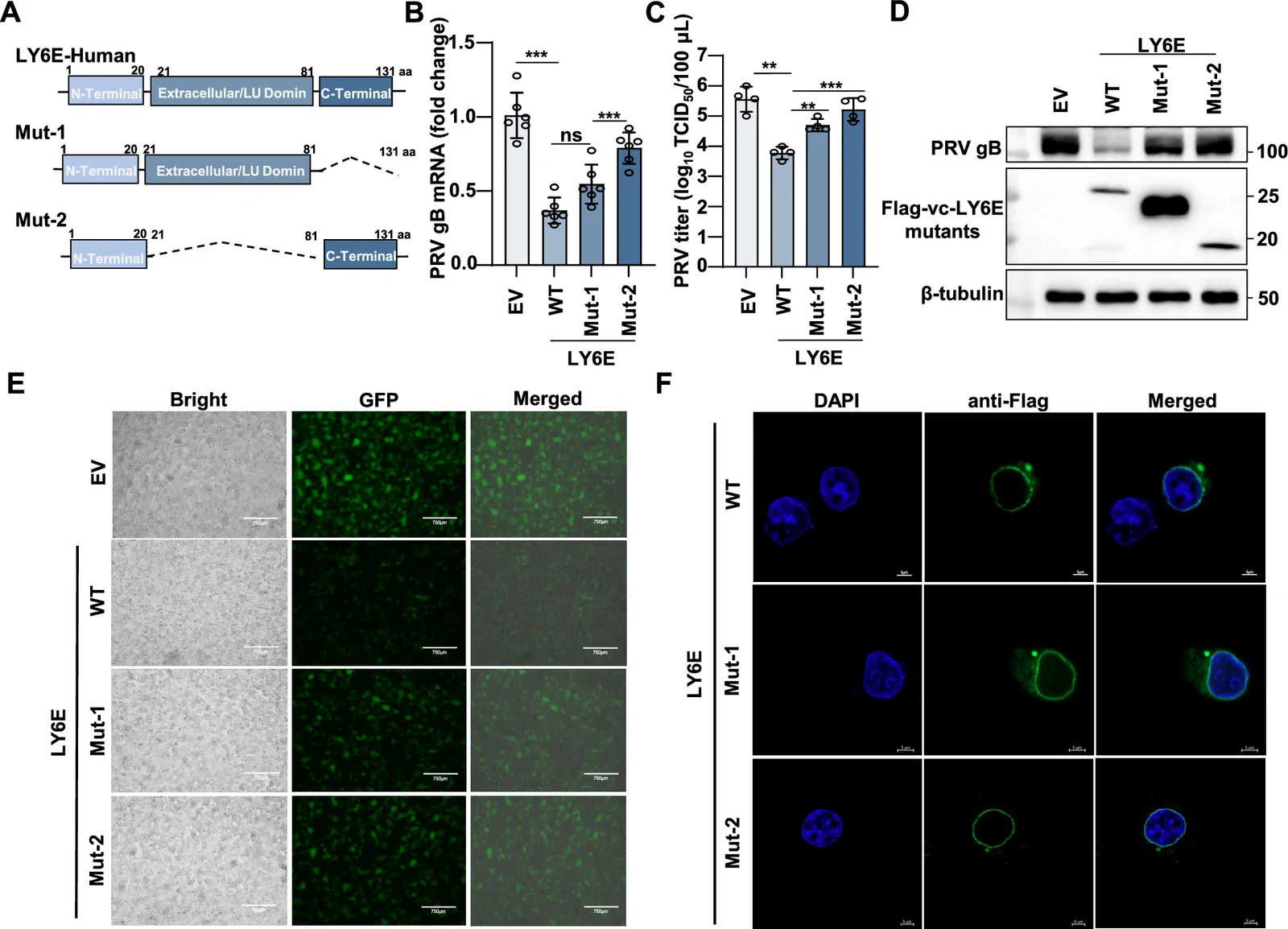
**Domain-specific contribution of lymphocyte antigen 6, family member E (LY6E) to antiviral activity against Pseudorabies virus (PRV) in HEK293T cells.** **A** Schematic representation of full-length human LY6E, and domain-specific mutant constructs ((Mut-1, Mut-2), indicating the extracellular/LU domain (absent in Mut-2), and the C-terminal region (deleted in Mut-1). **B** Quantitative reverse transciption PCR (RT-qPCR) of PRV gB messenger RNA (mRNA) levels in cells transfected with empty-vector (EV), wild-type LY6E (WT), or LY6E mutant constructs (Mut-1, Mut-2). **C** Viral titers in culture supernatants quantified by the 50% tissue culture infectious dose (TCID_50_). **D** Immunoblot analysis of expression of PRV-gB, Flag-tagged WT LY6E, or LY6E mutants; β-tubulin serves as a loading control. **E** Immunofluorescence analysis of PRV infection in cells transfected with EV, WT LY6E, or LY6E mutant constructs. Green fluorescent protein (GFP) signals indicate viral infection; bright-field images show cell morphology; and merged images show overlay of GFP and bright-field. **F** Confocal immunofluorescence analysis of Flag-tagged WT LY6E, MUT-1, and MUT-2. LY6E was detected using anti-Flag antibody (green), and nuclei were counterstained with DAPI (blue). Asterisks indicate a strong significant difference at **P < 0.001 and a very strong statistical difference at ***P < 0.0001; ns, not significant.

To further understand the structural factors required for the antiviral activity of LY6E, we investigated the functional contribution of the extracellular/LU domain and the C-terminal region of LY6E to PRV infection. The extracellular/LU domain of LY6E serves as a conserved functional domain of LY6E, while the C-terminal region is important for the localization of LY6E on the cell membrane. We generated two mutants of LY6E: the C-terminal was deleted in Mut-1, and the extracellular/LU domain was absent in Mut-2. The antiviral potency of these mutants was compared with that of full-length WT LY6E (Figure 3A). RT-qPCR analysis showed that WT LY6E obviously reduced PRV-gB mRNA levels relative to the EV control, whereas both Mut-1 and Mut-2 exhibited partial loss of antiviral efficacy (Figure 3B). Consistently, viral titer quantification using the TCID₅₀ assay demonstrated that WT LY6E-expressing cells showed a significant reduction in infectious PRV production; in contrast, cells transfected with either domain-deletion mutant (Mut-1 or Mut-2) yielded significantly higher viral titers, failing to achieve the level of antiviral restriction observed with full-length WT LY6E. In fact, both domain-specific mutants possessed partial antiviral activity compared to the WT LY6E (Figure 3C). Taken together, these findings indicate that LY6E requires both domains for maximal antiviral activity. These findings were further validated by immunoblot analysis, which revealed a strong suppression of PRV-gB protein levels in WT LY6E-expressing cells, while inhibition of viral protein was less noticeable in Mut-1 and Mut-2 as compared to the EV control (Figure 3D). We also performed live-cell imaging using a fluorescence microscope. The decrease in GFP-positive signals in WT LY6E-expressing cells indicated successful suppression of viral infection relative to the EV control. In contrast, the domain-mutant constructs (Mut-1 or Mut-2) of LY6E-expressing cells did not achieve complete suppression of PRV infection and showed higher GFP fluorescence than WT LY6E (Figure 3E). Additionally, confocal microscopy was performed to examine the effect of LY6E deletion mutants on subcellular localization (Figure 3F). WT LY6E, Mut-1, and Mut-2 exhibited membrane-associated and intracellular localization patterns. Although Mut-1 exhibited staining patterns similar as WT LY6E, the present data are insufficient to draw any conclusion regarding the contribution of the C-terminal region to LY6E membrane targeting. In contrast, the extracellular/LU domain deletion mutant (Mut-2) displayed a localization pattern that was confined to the perinuclear region, with relatively less diffuse cytoplasmic distribution as compared to the WT LY6E. These observations suggest that deletion of the extracellular/LU domain may influence the intracellular distribution of LY6E; however, additional studies will be required to define the precise contribution of both domains to LY6E trafficking and membrane localization.

Collectively, these results suggested that both the extracellular/LU domain and the C-terminal region of LY6E are important for the antiviral function of LY6E. Loss of either domain caused partial loss of antiviral activity of LY6E against PRV, highlighting the significance of coordinated functional domains of LY6E.

### Transcriptomic analysis of PRV infection in LY6E-expressing HEK293T cell

**Figure 4:**
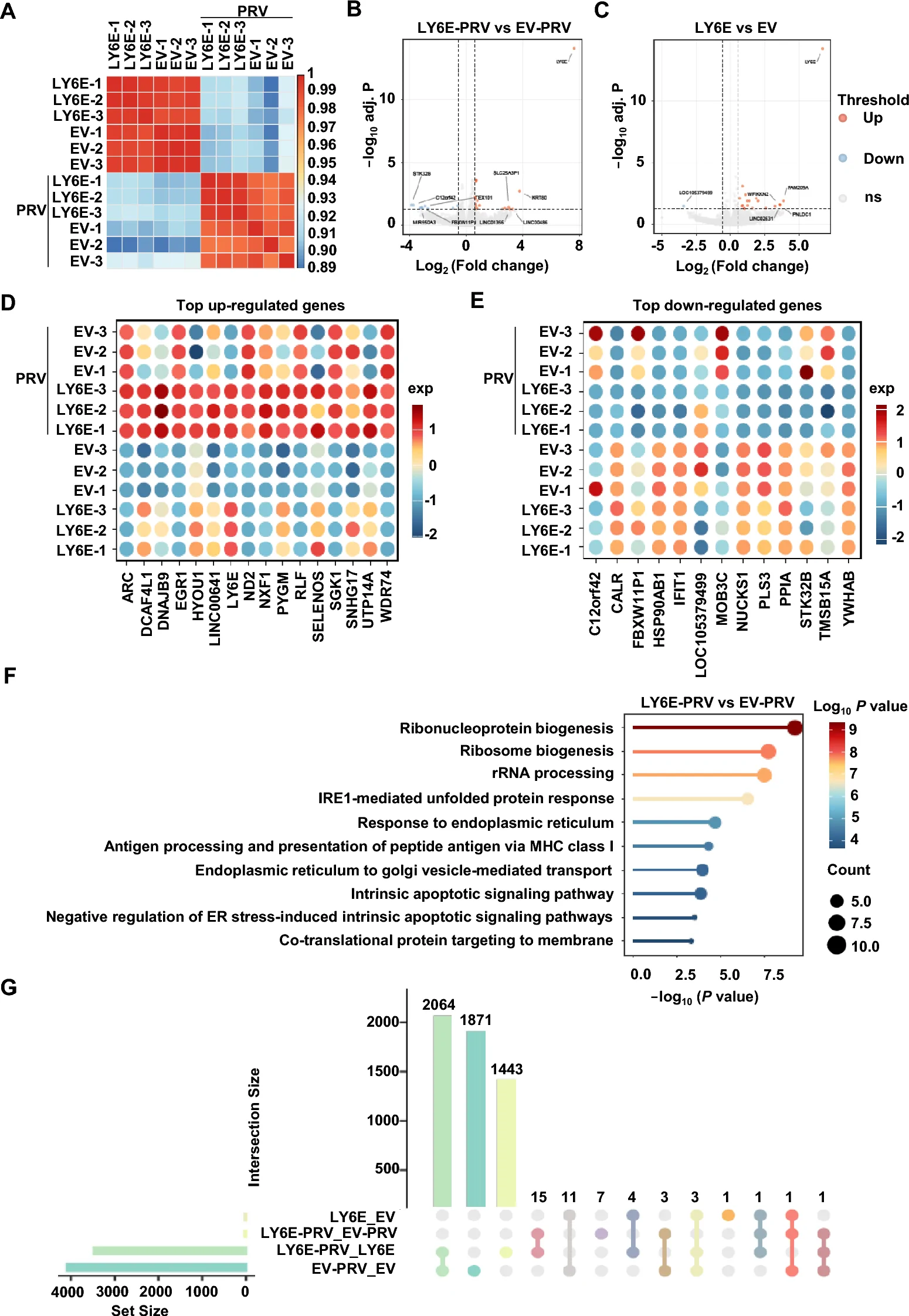
**Transcriptomic analysis of Pseudorabies virus (PRV) infection in HEK293T cell line expressing lymphocyte antigen 6, family member E (LY6E) or empty vector (EV)**. **A** Heat map analysis of biological replicates demonstrating high consistency of RNA sequencing (RNA-seq) data. **B** Volcano plot showing differentially expressed genes (DEGs) in PRV-infected LY6E-expressing (LY6E-PRV) cells vs. PRV-infected EV (EV-PRV) cells, highlighting upregulated (red) and downregulated (blue) genes in response to LY6E expression during PRV infection. **C** Volcano plot showing DEGs in LY6E vs. EV highlighting upregulated (red) and downregulated (blue) genes, illustrating the transcriptional effect of LY6E in the absence of PRV infection. **D, E** Heat map of top upregulated and downregulated genes identified in LY6E and EV during PRV infection. **F** Gene ontology (GO) pathway analysis for LY6E-PRV vs. EV-PRV cells, highlighting significantly enriched biological processes associated with LY6E-mediated modulation during PRV infection. **G** UpSet plot showing overlaps and distribution of DEGs among four comparisons, illustrating shared and comparison-specific transcriptional responses.

To investigate how LY6E remodels host transcriptional programs upon PRV infection, we performed RNA-seq in HEK293T cells expressing LY6E or EV, with or without PRV infection. More than 7,000 genes were identified, and strong Pearson correlations between biological replicates confirmed data quality. Uninfected and PRV-infected samples formed distinct clusters, indicating that PRV infection causes transcriptional changes (Figure 4A). Consistent with this notion, thousands of differentially expressed genes (DEGs) were observed in both the EV- and LY6E-expressing cells during infection (Additional file 6). To specifically define LY6E-dependent transcriptional changes during PRV infection, we focused our subsequent analysis on the comparison between PRV-infected LY6E-expressing cells and PRV-infected EV cells (LY6E-PRV vs. EV-PRV). The volcano plot revealed only a limited set of statistically significant DEGs between these two infected groups, which indicates that LY6E does not elicit widespread transcriptional perturbation in the context of PRV infection (Figure 4B). Similarly, ectopic LY6E expression alone induced minimal transcriptional changes in uninfected cells (Figure 4C).

We next visualized DEGs derived from the LY6E-PRV versus EV-PRV comparison using heatmaps (Figure 4D, E). The significantly upregulated genes included DCAF4L1, DNAJB9, HYOU1, LINC00641, LY6E, NXF1, PYGM, SELENOS, and UTP14A, while the significantly downregulated genes comprised C12orf42, FBXW11P1, MOB3C, STK32B, and TMSB15A. Functional annotation showed that these DEGs were mainly associated with endoplasmic reticulum (ER) stress adaptation, protein quality control, RNA metabolism, and cellular regulatory pathways, suggesting that LY6E selectively influences host homeostasis-related processes during PRV infection.

The Gene Ontology (GO) analysis of DEGs identified in the LY6E-PRV versus EV-PRV comparison revealed the enrichment of pathways related to ribosome biogenesis, ribosomal RNA (rRNA) processing, ribonucleoprotein complex biogenesis, IRE1-mediated ER stress responses, antigen processing and presentation via major histocompatibility complex (MHC) class I molecules, and regulation of intrinsic apoptotic signaling (Figure 4F). These findings suggest that LY6E selectively modulates host pathways involved in protein homeostasis, RNA metabolism, antigen presentation, and cellular stress adaptation upon PRV infection. In parallel, comparison between uninfected LY6E and EV cells identified only a limited number of DEGs and modest enrichment of homeostasis-related pathways (Additional file 6), providing supporting evidence that LY6E overexpression alone has minimal impact on basal transcription. An UpSet plot further illustrated that the transcriptional shifts provoked by PRV infection were far more extensive than the LY6E-associated transcriptional changes (Figure 4G), confirming PRV infection as the dominant driver of host transcriptomic remodeling.

Collectively, these results demonstrate that PRV infection drives extensive alterations in host gene expression. During PRV infection, LY6E selectively modulates pathways governing protein synthesis and quality control, antigen presentation, ER stress responses, and apoptotic regulation, suggesting that LY6E fine-tunes the host cellular environment to influence infection outcome.

### LY6E impairs internalization by interacting with PRV glycoproteins

**Figure 5:**
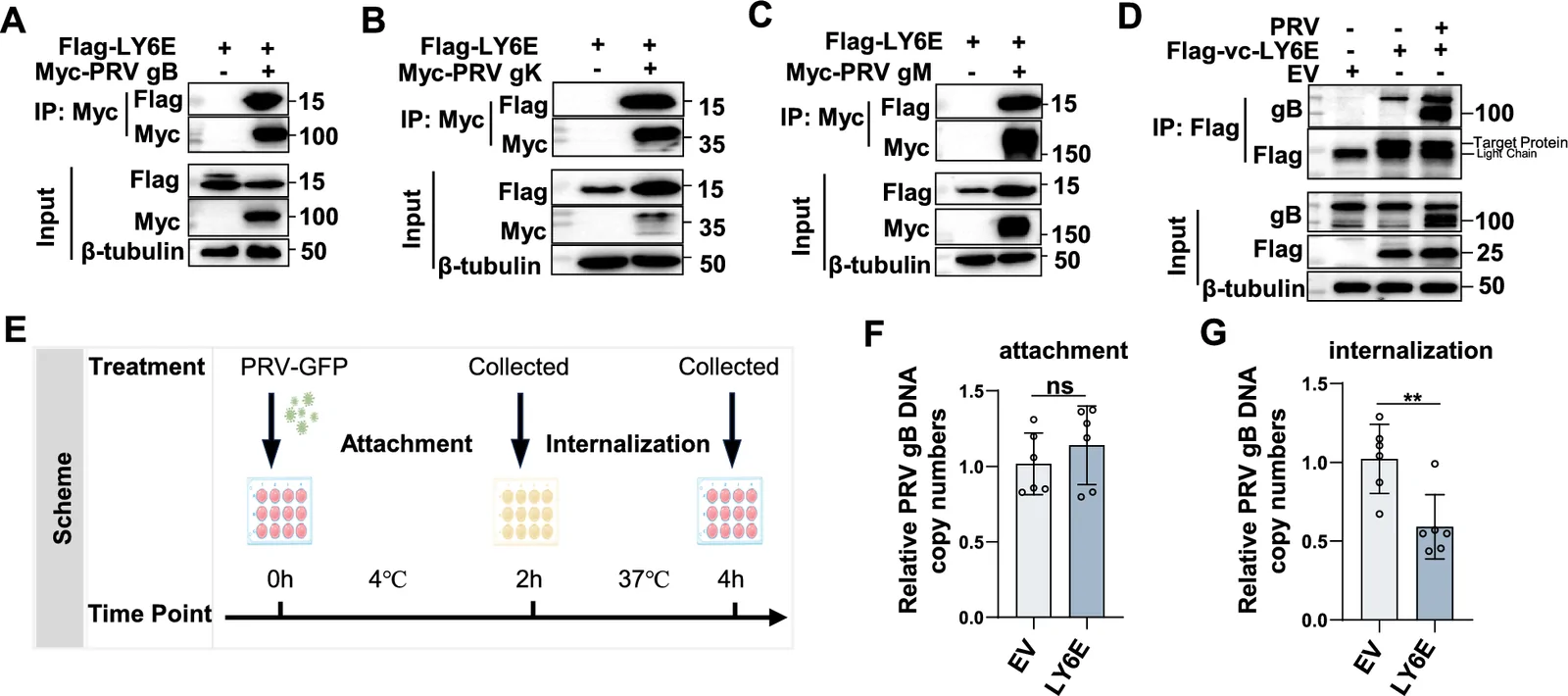
**LY6E interacts with Pseudorabies virus (PRV) glycoproteins and restricts viral internalization.** **A–C** Co-immunoprecipitation analysis of LY6E interactions with PRV glycoproteins. HEK293T cells were co-transfected with Flag-tagged LY6E and Myc-tagged PRV glycoproteins gB (**A**), gK (**B**), and gM (**C**). Cell lysates were immunoprecipitated using anti-Myc beads followed by immunoblotting with anti-Myc or anti-Flag antibodies. Input control show protein expression levels, and with β-tubulin serves as a loading control. **D** Co-immunoprecipitation analysis of protein interactions in HEK293T cells transfected with the empty vector (EV) control, LY6E, and LY6E followed by PRV infection. Cell lysates were immunoprecipitated using anti-Flag beads followed by immunoblotting with anti-Flag or anti-PRV-gB antibodies. Input control show protein expression levels; β-tubulin serves as a loading control. **E** Schematic representation of the PRV attachment and internalization assay. A549 cells expressing EV or LY6E were incubated with PRV-green fluorescent protein (GFP) at 4 ℃ to allow viral attachment, followed by incubation at 37 ℃ to allow viral internalization. Samples were collected at the indicated time points. **F, G** Quantification of PRV genomic DNA during viral attachment (**F**) and internalization (**G**) by quantitative (q) PCR. LY6E expression did not affect viral attachment but significantly reduced viral internalization. Asterisks indicate a strong significant difference at **P < 0.001; ns, not statistically significant.

Based on the aforementioned results, we hypothesize that LY6E restricts PRV infection by interfering with early entry events in the viral replication cycle. Therefore, we first explored the mechanistic physical interaction between LY6E and viral structural proteins implicated in PRV entry. HEK293T cells were co-transfected with Flag-tagged LY6E and Myc-tagged PRV glycoproteins including gB, gC, gD, gE, gG, gH, gI, gK, gL, and gM. Co-immunoprecipitation assays were performed using anti-Myc magnetic beads, with results verified by immunoblotting. Our findings demonstrated that LY6E formed complexes with multiple PRV envelopeglycoproteins associated with viral entry and spread, including gB, gK, and gM (Figure 5A–C). In contrast, no interaction was observed between LY6E and gC, gD, gE, gG, gH, gI, and gL glycoproteins under the same experimental conditions (Additional file 7). To assess whether LY6E interacts with PRV glycoproteins during infection, co-immunoprecipitation was performed in LY6E-expressing cells infected with PRV. PRV-gB was detected in LY6E immunoprecipitates, indicating that this interaction occurs in the context of viral infection (Figure 5D).

Given the involvement of these glycoproteins in distinct steps of viral entry and spread, we next evaluated whether LY6E affects PRV attachment or internalization. A stepwise entry assay based on temperature control was used to functionally separate these two processes, as schematically illustrated in Figure 5E. In this assay, viral attachment was allowed at 4 ℃, a condition that permits binding but prevents membrane fusion and uptake, followed by a temperature shift to 37 ℃ to initiate viral internalization. LY6E did not impair viral attachment, as quantified by PRV genomic DNA levels. At the viral attachment phase of PRV, there was no significant difference observed between LY6E-expressing cells and the EV control (Figure 5F). In contrast, LY6E expression led to a significant decrease in internalized PRV genomes compared with the EV control when the temperature was shifted to 37 ℃ (Figure 5G). These results indicate that LY6E specifically inhibits a subsequent post-attachment step of PRV entry without disrupting the early stage of viral attachment to host cells.

## Discussion

LY6E was initially characterized as a GPI-anchored cell surface protein, serving as a developmental marker for thymocytes. Its functional role was gradually extended from T-cell activator to a driver of ISG expression, with a major influence on viral pathogenesis [8]. Notably, LY6E was previously reported to exert proviral effects in certain RNA virus infections, thereby facilitating CD4-dependent HIV-1 entry and post-entry uncoating of influenza A virus, and boosting flavivirus infection [8, 22, 24]. Our study highlights LY6E as a potent restriction factor against PRV. It provides new insight into antiviral role of LY6E through its interaction with PRV glycoproteins associated with viral replication.

In our study, LY6E is highlighted as a restriction factor against PRV in both human and porcine cells. Reciprocal phenotypes from gain- and loss-of-function experiments demonstrate that LY6E overexpression is associated with reduced PRV protein levels and viral titers, whereas a considerable reduction in LY6E mRNA expression leads to enhanced viral replication, providing strong evidence for LY6E-mediated antiviral restriction of PRV infection (Figure 1). The antiviral role of LY6E is further supported by the dose-dependent and time-dependent inhibitory effects on PRV infection observed in this study. Our findings align with those of Zhao et al. [16], who reported that LY6E expression inhibits coronavirus (HCoV-OC43) entry in multiple human cell lines. However, other studies have demonstrated LY6E as a proviral factor for HIV-1 entry in a cellular context [24, 25]. Together, these observations highlight that LY6E functions as a regulator of viral infection with antiviral or proviral activity depending on the viral system. In our study, LY6E clearly acts as a restriction factor against PRV and HSV-1.

In addition to functional antiviral assays, our transcriptome data provide insights into the LY6E-dependent host response to PRV infection (Figure 4). LY6E by itself has minimal impact on basal gene expression, which is consistent with its known function as a membrane-associated modulator rather than a transcription factor. PRV itself induces global alterations in host gene expression; however, in the context of PRV infection, LY6E is associated with subtle yet consistent changes in specific host pathways, including ER stress, unfolded protein response, RNA–protein machinery, and antigen presentation. These pathways are typically activated during viral infection for cellular adaptation to cope with the high demand for viral protein synthesis and viral antigen for immune surveillance. Notably, PRV downregulates ISGs such as IFIT1 and IFIT3, suggesting  that antagonism of IFN signaling is an immune evasion tactic employed by various viruses. These findings align with a previous study demonstrating that PRV suppresses type I and type III IFN signaling by proteasomal degradation of the Janus kinase (JAK1) and tyrosine kinase 2 (TYK2) [26]. ER stress-related pathways are also enriched in our transcriptome analysis, consistent with prior findings that PRV infection disrupts ER homeostasis and triggers the unfolded protein response [27]. The entry of alphaherpesviruses involves strongly coordinated membrane remodeling and cytoskeleton-dependent trafficking to facilitate viral uptake [28]. LY6E, as a GPI-anchored protein enriched in membrane microdomains, influences these processes and may interfere with PRV entry by altering membrane microdomain organization and associated cytoskeletal interactions. Furthermore, our transcriptome data revealed upregulated host genes related to cytoskeletal organization and cellular stress response, supporting the notion that LY6E modulates host cellular architecture rather than directly inhibiting viral gene expression. These findings are consistent with those reported in previous studies that highlight LY6E as a membrane-associated regulator of viral entry rather than a direct transcriptional effector [23]. Overall, our findings demonstrate that during PRV infection, LY6E is associated with smaller and targeted alterations in specific cellular processes, including stress responses, immune function, and host–virus interactions, rather than causing broad alterations in global gene expression.

Mutational and domain deletion studies of LY6E elucidate the mechanistic insight into its role as a restriction factor in PRV infection (Figures 2, 3). Our study does not align with the LY6E structural–functional model established in the coronavirus system. In accordance with previous studies, GPI-dependent membrane localization through the N99 residue and evolutionarily conserved L36 residue play a vital role in LY6E activity to modulate viral fusion and entry during infection by human coronaviruses and influenza A virus [16, 22, 23]. Our mutagenesis studies (L36A/N99A) demonstrate that the same structural features of LY6E are not strictly required for PRV restriction, as disruption of these residues led to non-significant loss of LY6E-mediated antiviral activity. Consistently, the confocal analysis revealed changes in the subcellular distribution of LY6E in the mutant construct. These results demonstrate that L36 and N99 may not exclusively regulate antiviral function of LY6E. Similarly, partial loss of the antiviral function of LY6E against PRV was observed in the domain-deletion analysis. Deletion of either the extracellular/LU domain or the C-terminal region led to a reduction but did not fully abolish PRV restriction. Interestingly, deletion of either domain displayed differential effects on the localization of LY6E. Although the C-terminal deletion mutant displayed a similar localization pattern as the WT LY6E, the confocal results alone in the present study are insufficient to determine subcellular localization, as the GPI anchor is contained at the C-terminus, which may disturb attachment of LY6E to the membrane; in comparison, the extracellular/LU domain led to restricted localization at the nuclear membrane. These observations suggest that the extracellular/LU domain may contribute to proper intracellular distribution of or trafficking of LY6E (Figure 3). These findings may support the combined function of both domains of LY6E to interfere with PRV entry. Notably, previous studies focused on individual residues required for viral entry and LY6E localization [16, 23]. Our findings address the previously unexplored structural–functional aspects of LY6E by systematic analysis of the extracellular/LU domain and the C-terminal contributions using PRV phenotyping. Collectively, these findings broaden the entry-mediated restriction model from RNA viruses to the DNA alphaherpesvirus system, in which conserved residues, both functional domains, and correct localization govern the proper antiviral activity of LY6E.

Consistent with previous research identifying LY6E as a membrane-associated regulator of viral entry [22], the present study demonstrates that LY6E restricts PRV infection at the post-attachment stage. Mechanistically consistent with our findings, prior studies have reported that LY6E blocks spike-mediated membrane fusion of coronaviruses without interfering with viral binding [16, 23], whereas it facilitates the post-attachment uncoating of influenza A virus [22]. Extending these findings to alphaherpesviruses, we demonstrate that LY6E impairs viral internalization and associates with PRV glycoproteins. Together, these results identify LY6E as a restriction factor against PRV and expand its antiviral spectrum to DNA viruses.

While our attachment and internalization assays confirm that LY6E interferes with early PRV entry, the exact subcellular sites driving this antiviral activity remain incompletely resolved. Cellular fractionation assays indicate that LY6E localizes to both the cell membrane and cytosol. Meanwhile, co-IP-detected PRV glycoproteins likely include immature isoforms undergoing biosynthesis and secretory pathway processing, and transcriptomic analysis further revealed enrichment of ER-associated biological processes. These findings suggest that, beyond blocking viral internalization, intracellular LY6E may modulate the processing and trafficking of viral glycoproteins. Collectively, our results validate the interaction between LY6E and PRV glycoproteins but cannot discriminate whether this association occurs predominantly during plasma membrane-localized viral entry, intracellular glycoprotein maturation, or a combination of both processes. Future investigations are therefore required to dissect the individual contributions of these membrane-related mechanisms to LY6E-mediated PRV restriction.

## Conclusion

Our study establishes LY6E as a host factor that inhibits PRV infection by impairing post-attachment entry, while defining the key viral structural components underlying this activity. Structural, functional, and transcriptomic analyses support a model in which LY6E reshapes host cellular processes to restrict viral entry and early infection. These findings thus illuminate the entry-based antiviral function of LY6E in the herpesvirus family. Although our work elucidates the mechanical aspects of LY6E function, future investigations can be carried out on IFN-mediated LY6E regulation to control PRV infection, and in vivo studies required to understand the deep mechanism of LY6E. These studies will establish a scientific basis for therapeutic intervention to control viral diseases.

## Supplementary Information

**Additional file 1. Primers used for LY6E mutagenesis.** List of primer sequences used for site-specific mutagenesis of LY6E including primers for the L36A and N99A substitutions and deletion constructs targeting extracellular domain and C-terminal regions.

**Additional file 2. Primer sequences used for RT-qPCR.** List of primer sequences used for RT-qPCR analysis, including primers for human and mouse *LY6E*, *ISG-15*, *ISG-20*, and *GAPDH*, PRV *UL27-gB*, and HSV-1 *gB*.

**Additional file 3. Temporal expression of** ***LY6E, ISG-15,* and** ***ISG-20* in iBMDMs following IFN-β stimulation.** RT-qPCR analysis showing the temporal expression of *LY6E*, *ISG-15*, and *ISG-20* in immortalized bone marrow-derived macrophages (iBMDMs) following IFN-β-stimulation at 0, 6, 12, and 24 h compared with mock-untreated controls.

**Additional file 4. Transfection efficiency in different cell lines measured by GFP expression. **Flow cytometric analysis of GFP-positive cells showing transfection efficiency of a GFP-expressing plasmid in HEK293T, A549, PVEC, and PK-15 cells using 200 and 400 ng of plasmid with empty vector (EV) as a control.

**Additional file 5. Subcellular fractionation analysis of N-terminally Flag-tagged LY6E in HeLa cells.** Immunoblotting and densitometric analysis of membrane and cytosolic fractions from HeLa cells expressing N-terminally Flag-tagged LY6E, demonstrating the relative distribution of LY6E using ATP1A1 and β-tubulin as membrane and cytosolic fraction makers, respectively.

**Additional file 6. Transcriptomic analysis of PRV infection in HEK293T cell line expressing LY6E or empty vector (EV).** Comparative transcriptomic analysis of PRV-infected or uninfected HEK-293T cells expressing LY6E or empty vector (EV), showing differentially expressed genes (DEGs) by volcano plots, and Gene Ontology (GO) enrichment analysis of identified DEGs.

**Additional file 7. Screening of interactions between LY6E and PRV glycoproteins. **Co-immunoprecipitation analysis screening the interaction of Flag-tagged LY6E with Myc-tagged PRV glycoproteins gB, gC, gD, gE, gG, gH, gI, gK, gL, and gM in HEK293T cells.

## Data Availability

The data that support the findings of this study are available from the corresponding author upon reasonable request.
