# Supplementary material for: LY6E regulates Pseudorabies virus early infection via impairing viral internalization
Source: Vet Res. 2026 Sep 12;57:164. doi: 10.1186/s13567-026-01835-6 (PMC13570520; doi:10.1186/s13567-026-01835-6)
Supplement: Supplementary file 1 [file 13567_2026_1835_MOESM1_ESM.docx]

**Additional file 1. Primers used for LY6E mutagenesis**

| **Primer name** | **Sequence (5´-3´)** |
| --- | --- |
| L36A-F | ATCTGTACTGCGCGAAGCCGACCATCTGCTCCG |
| L36A-R | CTTCGCGCAGTACAGATTGCTCTTCTGGTTCA |
| N99A-F | TCTGTGCGCTTTCAGTGCGGCCGATGGCGGGC |
| N99A-R | CACTGAAAGCGCACAGAAAGCTCTGGCAGCAG |
| loss-ECD-F | TGGAGCGAGCCTTCAGTGCGGCCGATGGCGGGC |
| loss-ECD-R | CACTGAAGGCTCGCTCCACACCCAGAAGGGCAG |
| loss-C-terminal-F | CTGTGCAATCATATGGACTACAAAGACCATGACG |
| loss-C-terminal-R | GTCCATATGATTGCACAGAAAGCTCTGGCAGCA |
