# Supplementary material for: LY6E regulates Pseudorabies virus early infection via impairing viral internalization
Source: Vet Res. 2026 Sep 12;57:164. doi: 10.1186/s13567-026-01835-6 (PMC13570520; doi:10.1186/s13567-026-01835-6)
Supplement: Supplementary file 2 [file 13567_2026_1835_MOESM2_ESM.docx]

**Additional file 2. Primer sequences used for RT-qPCR**

| **Gene** | **Species** | **Primer name** | **Sequence (5´-3´)** |
| --- | --- | --- | --- |
| *LY6E* | Human | LY6E-F | CAGCTCGCTGATGTGCTTCT |
| *LY6E* | Human | LY6E-R | CAGACACAGTCACGCAGTAGT |
| *LY6E* | Mouse | LY6E-F | TGCGGGCTTTGGGAATGTC |
| *LY6E* | Mouse | LY6E-R | CGGATGCCACACCGAGATT |
| *PRV UL27-gB* | PRV | gB-F | AAGTTCAAGGCCCACATCTA |
| *PRV UL27-gB* | PRV | gB-R | TGAAGCGGTTCGTGATGG |
| *GAPDH* | Human | GAPDH-F | GAGTCAACGGATTTGGTCG |
| *GAPDH* | Human | GAPDH-R | TTGATTTTGGAGGGATCTCG |
| *GAPDH* | Mouse | GAPDH-F | AACTTTGGCATTGTGGAAGGGCTC |
| *GAPDH* | Mouse | GAPDH-R | TGGAAGAGTGGGAGTTGCTGTTGA |
| *ISG-15* | Mouse | ISG-15-F | GGTGTCCGTGACTAACTCCAT |
| *ISG-15* | Mouse | ISG-15-R | TGGAAAGGGTAAGACCGTCCT |
| *ISG-20* | Mouse | ISG-20-F | GACCCGAGGGAGAGATCAC |
| *ISG-20* | Mouse | ISG-20-R | CAGGGCATTGAAGTCGTGCT |
| *HSV-1-gB* | HSV-1 | gB-F | AACGCGACGCACATCAAG |
| *HSV-1-gB* | HSV-1 | gB-R | CTGGTACGCGATCAGAAAGC |
