# Supplementary material for: LY6E regulates Pseudorabies virus early infection via impairing viral internalization
Source: Vet Res. 2026 Sep 12;57:164. doi: 10.1186/s13567-026-01835-6 (PMC13570520; doi:10.1186/s13567-026-01835-6)
Supplement: Supplementary file 3 [file 13567_2026_1835_MOESM3_ESM.docx]

**Additional file 3.** Temporal expression of *LY6E, ISG-15*, and *ISG-20* in iBMDM following IFN-β stimulation.

(A-C) Quantitative RT-qPCR analysis of *LY6E* (A), *ISG-15* (B), and *ISG-20* (C) levels in immortalized bone marrow-derived macrophages (iBMDMs) treated with IFN-β for the indicated time points (3, 6, 12, 24 h). Mock (untreated) cells served as control. Data are presented as mean ± SEM from at least three independent biological replicates. Statistical significance was determined using Student’s *t*-test with **P <* 0.05*; **P <* 0.01*; ***P <* 0.001, *****P <* 0.0001.
