# Supplementary material for: LY6E regulates Pseudorabies virus early infection via impairing viral internalization
Source: Vet Res. 2026 Sep 12;57:164. doi: 10.1186/s13567-026-01835-6 (PMC13570520; doi:10.1186/s13567-026-01835-6)
Supplement: Supplementary file 4 [file 13567_2026_1835_MOESM4_ESM.docx]

**Additional file 4. Transfection efficiency in different cell lines measured by GFP expression.**

HEK293T, A549, PVEC, and PK-15 cells were transfected with increasing amounts (200 ng and 400 ng) of a GFP-expressing plasmid or empty vector (EV). 24 h post-transfection, GFP-positive cells were quantified by flow cytometry. Data are presented as percentage (%) of GFP-positive cells. Human-derived cell lines (HEK293T, A549) exhibited higher transfection efficiency than porcine-derived cell lines (PVEC, PK-15).
