# Supplementary material for: LY6E regulates Pseudorabies virus early infection via impairing viral internalization
Source: Vet Res. 2026 Sep 12;57:164. doi: 10.1186/s13567-026-01835-6 (PMC13570520; doi:10.1186/s13567-026-01835-6)
Supplement: Supplementary file 5 [file 13567_2026_1835_MOESM5_ESM.docx]

**Additional file 5. Subcellular fractionation analysis of N-terminally Flag-tagged LY6E in HeLa cells.**

**(A)** HeLa cells were transfected with either empty vector (EV) or Flag-tagged WT-LY6E. Whole-cell lysate (WCL), membrane fractions, and cytosolic fractions were prepared and analyzed by immunoblotting with labeled antibodies. ATP1A1 and β-tubulin were used as membrane and cytosolic markers, respectively. N-terminally Flag-tagged LY6E was predominantly detected in the membrane fraction, with lower levels in the cytosolic fraction, supporting its membrane-associated localization **(B)** Quantification of LY6E distribution relative to whole-cell lysate (WCL). Relative Flag-LY6E signals in membrane and cytosolic fractions were quantified by densitometric analysis using ImageJ and normalized to the corresponding Flag-LY6E signal in WCL. Data presented as relative intensity ratios, indicating the distribution of LY6E between membrane and cytosolic fractions. **(C)** Quantification of Flag-LY6E enrichment relative to fraction-specific markers. Flag-LY6E signal in the membrane and cytosolic fractions was quantified by densitometric analysis using ImageJ and normalized to ATP1A1 and β-tubulin, respectively. Data are presented as relative intensity ratios, demonstrating enrichment of LY6E in the membrane fraction relative to the cytosolic fraction.
