# Supplementary material for: LY6E regulates Pseudorabies virus early infection via impairing viral internalization
Source: Vet Res. 2026 Sep 12;57:164. doi: 10.1186/s13567-026-01835-6 (PMC13570520; doi:10.1186/s13567-026-01835-6)
Supplement: Supplementary file 6 [file 13567_2026_1835_MOESM6_ESM.docx]

**Additional file 6. Transcriptomic analysis of PRV infection in HEK293T cell line expressing LY6E or empty vector** **(EV)**

(A-B) Volcano plot showing differentially expressed genes (DEGs) in LY6E-PRV vs LY6E or EV-PRV vs EV comparisons (C-E) Gene Ontology (GO) enrichment analysis of DEGs in LY6E-PRV vs LY6E, EV-PRV vs EV, and LY6E vs EV comparisons.
