# Supplementary material for: LY6E regulates Pseudorabies virus early infection via impairing viral internalization
Source: Vet Res. 2026 Sep 12;57:164. doi: 10.1186/s13567-026-01835-6 (PMC13570520; doi:10.1186/s13567-026-01835-6)
Supplement: Supplementary file 7 [file 13567_2026_1835_MOESM7_ESM.docx]

**Additional file 7. Screening of interactions between LY6E and PRV glycoproteins**

Co-immunoprecipitation analysis of interactions between LY6E and PRV glycoproteins. HEK293T cells were co-transfected with Flag-tagged LY6E and Myc-tagged PRV glycoproteins gB, gC, gD, gE, gG, gH, gI, gK, gL, gM. Cell lysates were immunoprecipitated using anti-Myc beads followed by immunoblotting with anti-Myc and anti-Flag antibodies. Input samples were used to confirm expression, with β-tubulin serving as a loading control.
